# For Baldness

**Published:** 1880-02

**Authors:** 


For Baldness.—The following is highly recommended by Professor Kaposi :

℞.—Saponis viridis (German), alcoholis. aa. f℥ij
Solve, filtra, et adde ol. lavandulæ. . gtt.xx.–xxx

Pour one or two tablespoonsful upon the scalp ; then pour on a little water: rub smartly with the fingers, thus producing a copious lather. After four or five minutes' shampooing this way, rinse the head thoroughly with a towel. Then apply a little cosmoline. This process causes the hair to fall out in great abundance at first, but a new and fine growth of hair soon follows.—*New York Medical Journal.*

